# Comparison of the active and resident community of a coastal microbial mat

**DOI:** 10.1038/s41598-017-03095-z

**Published:** 2017-06-07

**Authors:** Daniela Clara Cardoso, Anna Sandionigi, Mariana Silvia Cretoiu, Maurizio Casiraghi, Lucas Stal, Henk Bolhuis

**Affiliations:** 1 Department of Marine Microbiology and Biogeochemistry, Royal Netherlands Institute for Sea Research, and Utrecht University, Den Hoorn, The Netherlands; 2 Department of Biotechnology and Biosciences, University of Milan-Bicocca, Milan, Italy; 3 Department of Freshwater and Marine Ecology, IBED, University of Amsterdam, Amsterdam, The Netherlands

## Abstract

Coastal microbial mats form a nearly closed micro-scale ecosystem harboring a complex microbial community. Previous DNA based analysis did not necessarily provide information about the active fraction of the microbial community because it includes dormant, inactive cells as well as a potential stable pool of extracellular DNA. Here we focused on the active microbial community by comparing 16S rRNA sequences obtained from the ribosomal RNA pool with gene sequences obtained from the DNA fraction. In addition, we aimed to establish an optimal and feasible sampling protocol that takes potential spatial and temporal heterogeneity into account. The coastal microbial mat investigated here was sampled randomly and at regular time points during one 24-h period. DNA and RNA was extracted and after conversion of the RNA fraction to cDNA, the V1-V3 and the V3-V4 regions of the 16S rRNA gene were targeted for high-throughput amplicon sequencing. We show that the community composition varies little in time and space whereas two amplified 16S regions gave significant different results. The largest differences were found when comparing the “resident community” (DNA) with the “active community” (cDNA/RNA); in the latter, Cyanobacteria dominated for almost 95% while they represented 60% of the resident fraction.

## Introduction

Coastal microbial mats consist of various, functional diverse microorganisms entangled in a sandy matrix that functions as a cooperative consortium^1, 2^. This consortium of microorganisms coordinates growth and nutrient cycling in a mutualistic way where each individual population on their own would be less successful under the same conditions^3^. Especially the primary producers in these mats, Cyanobacteria and diatoms, produce large amounts of extracellular polymeric substances (EPS) that stabilize the sediment and provide in interplay with the other mat inhabitants a nutritious environment for salt marsh vegetation^1^. Hence, these coastal microbial mats lay the foundation for a natural buffer zone between land and sea^4^. For the past 50 years coastal microbial mats have been extensively studied using a variety of techniques and approaches including microscopy analysis^5^, biochemical studies based on lipid content^6, 7^, analysis of carbohydrates and proteins^8^ and cultivation of microorganisms^9, 10^. Traditional culturing techniques are hampered by the inability to culture an important fraction of the extant microbial diversity^11^. However, in the era of molecular microbial ecology uncultured species can be identified by their genetic information. Initially, Sanger-based DNA sequencing and molecular fingerprinting were the standard techniques but were operated at low throughput and revealed only the most abundant species^12^. Recent developments in high throughput sequencing and bioinformatics allow the analysis of microbial communities at high resolution and elucidate the rare biosphere^13^. The current standard in microbial diversity studies is high throughput amplicon sequencing using Illumina’s sequencing by synthesis technology^14^. Dedicated primers targeting one or two of the nine hypervariable regions in the 16S rRNA gene allow to uncover the near full diversity of bacteria and archaea in any microbial ecosystem^15^. A potential drawback of DNA-directed community analysis is that environmental samples also contain extracellular DNA pools^16^ and DNA from inactive, dormant cells, or non-resident organisms that do not contribute to the active community at the time of sampling^17, 18^. This may hamper the ultimate goal of translating genetic diversity into extant functional diversity. Probing the actual RNA pool, which for more than 90% consists of ribosomal RNA may provide a better strategy to predict actual ecosystem functioning. RNA is only stable in active cells^19^, because it is conducting metabolic processes, while potential extracellular RNA pools are rapidly degraded after cell death^20^. Therefore, RNA makes a better indicator for extant microbial activity than DNA^21^.

Coastal microbial mats located at the North Sea coast of the Dutch barrier island Schiermonnikoog were previously analyzed by 454 pyrosequencing of the 16S rRNA V6 region using a single sample per mat type and per season. This work yielded insight in one of the most diverse marine microbial ecosystems^22^. However, this study was limited to only one sample per microbial mat type per season and the analysis only covered a short ~60 nt hypervariable region of the 16S rRNA gene. While a certain level of micro-heterogeneity between spatial nearby samples was observed^23^, nothing is known about potential temporal heterogeneity in the rRNA pool composition during a full day and night cycle.

Here, a statistical relevant sampling strategy for long-term monitoring of the pioneering marine microbial mats was developed. Analysis included 1) temporal and spatial heterogeneity, 2) the extant (DNA) versus the active (RNA) diversity and 3) the diversity obtained by targeting two different 16S rRNA regions for amplicon sequencing.

## Results

### General sequence statistics

Coastal microbial mat samples were subjected to MiSeq Illumina amplicon sequencing focusing on different variable regions of the bacterial 16S rRNA gene: V1-V3 and V3-V4. Amplicon sequencing yielded between 66834 to 508866 reads per sample with an average of 197898 reads (Table 1). The combined V1-V3 dataset of the six DNA samples revealed 1255 different OTUs at 95% identity (varying between 909 and 1038 OTUs per sample). For cDNA we calculated in total 1020 different OTUs (between 435 and 648 OTUs per sample). For the V3-V4 region 1164 OTUs were identified at 95% identity in the DNA fraction (between 854 to 1073 per sample) and 1053 OTUs in the RNA fraction (varying between 558 and 917 per sample) (Table 1). 99.9% of all the reads clustered into 41% and 27% of the DNA derived OTUs from the V1-V3 and V3-V4 region, respectively. The remaining 0.1% of the reads (59% and 73% of the OTUs) consisted of reads with only a few copies, representing the rare microbial diversity. For the cDNA-derived sequences between 15 and 20% of the OTUs contained 99.9% of the total number of reads and between 80 and 85% of the OTUs represent the remaining 0.1% of the diversity for the V1-V3 and V3-V4 region respectively. Alpha-diversity estimates at the 95% OTU level revealed highest diversity in the DNA derived sequences (Shannon index: 4.6 for the V1-V3 region and 4.9 for the V3-V4 region; (Table 1)). Diversity in the cDNA derived dataset was lower (Shannon diversity: 4.2 for V1-V3 and 3.8 for V3-V4). With ~1300 versus ~1200 estimated OTUs, the Chao richness estimator at 95% sequence identity was also higher in the DNA derived dataset than in the cDNA derived dataset.

**Table 1:** Statistical analysis of sequencing data.

	DNA^1^		RNA^1^	
Primer set	V1–V3	V3–V4	V1–V3	V3–V4
Sequences that passed QC	640378	1072313	966219	2070641
# OTUs (95% identity)	1255 (±39)	1164 (±69.5)	1020 (±70)	1053 (±108)
# Dominant^2^ OTUs (99.9%)	348	482	207	163
# Rare^2^ OTUs (0.1%)	907	682	813	890
Shannon diversity (95% OTU)	4.6	4.9	4.2	3.8
Chao richness (95% OTU)	1392	1300	1228	1198

^1^Data presented is average of the six samples. ^2^Dominant OTUs are the number of OTUs to which 99.9% of all the sequence reads clustered whereas rare OTUs only contain the remaining 0.1% of the total number of reads.

#### Cluster analysis of spatial and temporal heterogeneity versus activity and choice of 16S-rRNA gene region

**Figure 1:**
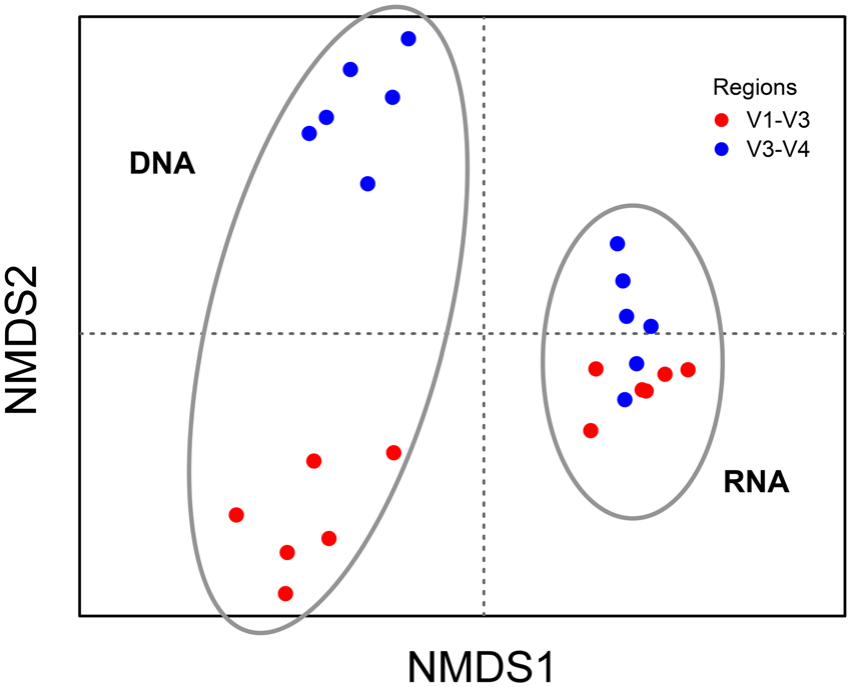
Non-metric multidimensional scaling plot (Bray-Curtis dissimilarity analysis) of the complete dataset (at genus level) including the six time points of sampling over a 24-h period, the different 16S rRNA regions analyzed and templates used (DNA versus RNA). Blue represents V3-V4 derived sequences and red represents V1-V3 derived sequences.

Our sampling strategy yielded six samples that represent both different time points and different parts of the microbial mat to include both temporal and spatial heterogeneity. To be able to directly compare the different datasets and infer their taxonomic identity, the OTUs at 95% identity were assigned up to the genus level. NMDS Bray-Curtis analysis of the observed community composition at this level revealed four distinct clusters with only minor overlap between the V1-V3 and the V3-V4 dataset of the cDNA fraction (Fig. 1). Using permutational multivariate analysis of variance (PERMANOVA) statistics with 1500 permutations revealed separate clustering of the nucleotide source (DNA vs cDNA: *p* ≤ 0.001) and of the targeted 16S rRNA gene region (*p*-value ≤ 0.01). Furthermore, PERMANOVA analysis revealed that although not identical (Fig. 1), samples taken at different time points or location within the 24-h sampling period clustered independent of time and space without significant difference (*p* 0.998).

#### Comparison of the 16S gene regions and the active bacterial community

**Figure 2:**
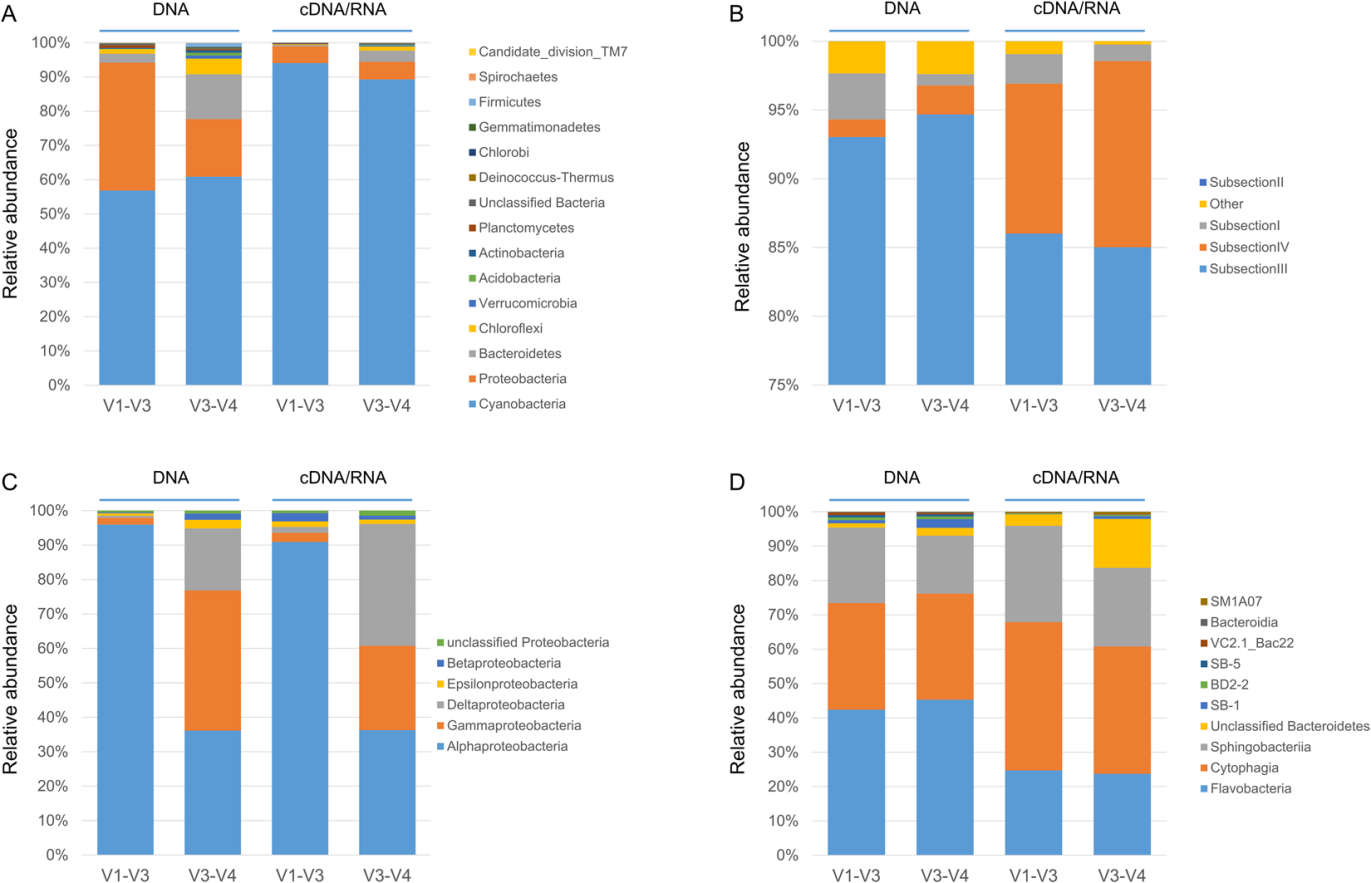
Taxonomic distribution comparison of the four different analysis. (**A**) bacterial phyla, (**B**) cyanobacterial order (**C**) proteobacterial class and (**D**) bacteroidetal class. “Other” represent sequence reads not further assigned within the taxa. The y axes in (**B**) starts at 75% for better representation of the distribution of the cyanobacterial orders. The nucleotide source is presented at the top whereas the amplified 16S region is presented in the x-axis. For large datasets only the dominant taxa were presented.

**Figure 3:**
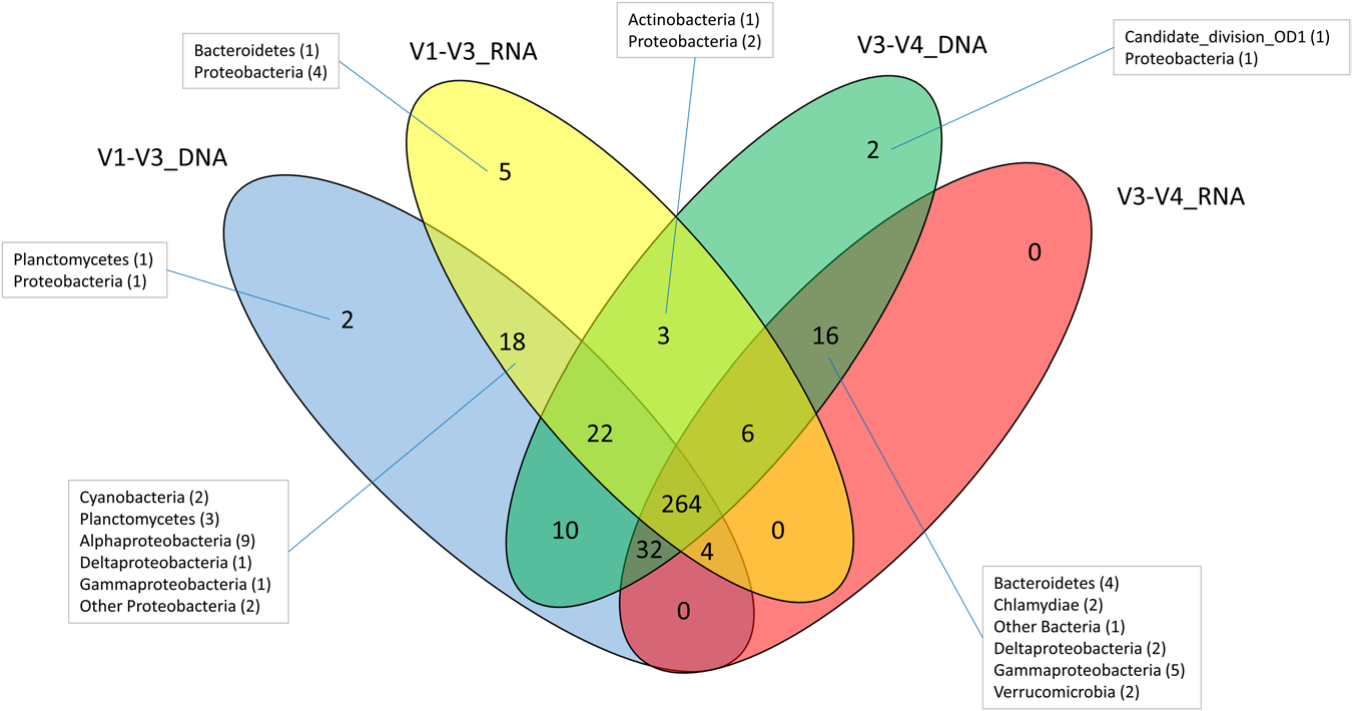
Venn diagram revealing the number of shared and unique genera in the different datasets. In total 384 different genera were identified in this analysis. Phyla unique to the different datasets are described in the outlined boxes with the number of different genera per phyla within brackets.

The taxonomic assignment of the V1-V3 and the V3-V4 16S rRNA sequences derived from the DNA template revealed Cyanobacteria (57% and 56%, respectively) and Proteobacteria (37% and 19%, respectively) as the dominant groups, while Bacteroidetes (3% and 15%, respectively) and Chloroflexi (1.4% and 5.5%, respectively) were among the less abundant phyla (Fig. 2). Particularly, some rare phyla were absent from the V1-V3 dataset (candidate division OD1, Chlamydiae and candidate division NPL-UPA2) relative to the V3-V4 dataset. In particular, this was the case in the RNA fraction that also lacked candidate divisions BD1-5, TM6, Hyd24-12 and the Fusobacteria in the V1-V3 dataset. At the genus level, the V1-V3 datasets of the DNA fraction revealed a lower diversity than the V3-V4 dataset, in agreement with the OTU diversity in Table 1. Sequences derived from the RNA template revealed a near absolute dominance of Cyanobacteria that made up 94% and 90% of the V1-V3 and the V3-V4 dataset, respectively, and Proteobacteria constituted 5%. Bacteroidetes and Chloroflexi were found at significant numbers in the V3-V4 dataset (3 and 1% respectively) but constituted less than 0.5% of the V1-V3 dataset. For the RNA fraction no significant difference was observed in Shannon diversity deduced from the genera distribution (data not shown). A Venn diagram was created revealing the number of unique and shared genera between the different nucleotide extraction methods and the analyzed 16S rRNA gene regions (Fig. 3). Out of the 384 identified genera, 69% (264) were shared by all sampling strategies, whereas only a few genera were unique to one of the sampling strategies. Unique to the V1-V3 dataset were 18 genera that were not found in the V3-V4 dataset. These genera included the Cyanobacteria genera, *Nostoc* and *Calothrix*, three Planctomycetes, while the majority were Alphaproteobacteria. Sixteen genera were unique to the V3-V4 dataset, and represented mainly Bacteroidetes and Proteobacteria (Fig. 3). Only a limited number of genera differed between the DNA and RNA fraction in either the V1-V3 or the V3-V4 dataset (7 and 2 genera, respectively). In terms of coverage, the number of different genera identified relative to the total number of genera found in this study, the V3-V4 primer set revealed only a slightly better coverage (92.5%) than the V1-V3 primer set (91.6%).

At the cyanobacterial order level (Fig. 2B) the largest variation was found when comparing the RNA with the DNA pool. This difference is most noticeable when looking at the contribution of the subsection IV in relation to subsection II. Subsection IV contributes about 10-fold more, in the RNA pool than subsection II. The choice of 16S rRNA gene region hardly affected this result. In contrast, the proteobacterial class shows a larger effect depending on the sequenced 16S rRNA region (Fig. 2C). While Alphaproteobacteria dominate in the DNA and RNA pools when the V1-V3 region was considered, these bacteria are at the second place according to the V3-V4 region. The latter region revealed a more even distribution between the three dominant proteobacterial classes. Gammaproteobacteria dominate the DNA fraction taking the V1-V3 region into account, while Deltaproteobacteria dominate in the RNA fraction when the V3-V4 region was considered. Class distribution within the third most abundant phyla of Bacteroidetes revealed only small differences between the different experimental approaches. There appeared to be a slightly higher contribution of Cytophaga in the RNA fraction of the V3-V4 region and a concomitant lower contribution of the Flavobacteria, while in the V3-V4/RNA derived sequences a group of unclassified Bacteroidetes increased in relative abundance.

When zooming in at the genus level, in both 16S rRNA regions an unassigned genus of the subsection III, family I Cyanobacteria dominated the extant diversity making up ~30% of the total bacterial community (Table 2). *Coleofasciculus* sp. took the second place with 6–9% in the DNA fraction and 16–20% in the RNA fraction. *Lyngbya* sp. was in the top 10 most abundant Cyanobacteria in the RNA fraction. The only other group of bacteria present in the top 10 of the RNA derived sequences was the deltaproteobacterium *Nannocystis*, comprising 0.5% of the total diversity. In contrast, amongst the Proteobacteria only alphaproteobacterial genera, mostly of the family of Rhodobacteraceae, were found in the DNA fraction (e.g. unassigned, *Loktanella*, and *Jannaschia*). Chloroflexi and Bacteroidetes were among the top 10 of most common organisms in the V3-V4/DNA dataset.

**Table 2:** Dominant OTUs at the genus level for both regions, V1-V3 and V3-V4 and for DNA and RNA.

V1V3–DNA	%	V1V3-RNA	%
Unass. genus 1 (Cyanobacteria, sub III, fam I)	32.7	Unass. genus 1 (Cyanobacteria, sub III, fam I)	29.1
Unass. genus 2 (Cyanobacteria, sub III, fam I)	8.3	*Coleofasciculus* (Cyanobacteria, sub III, fam I)	19.9
Unass. genus (Alphaproteobacteria, Rhodobacteraceae)	7.5	Unass. genus 2 (Cyanobacteria, sub III, fam I)	12.8
*Coleofasciculus* (Cyanobacteria, sub III, fam I)	6.3	*Lyngbya* (Cyanobacteria, sub III, fam I)	10.7
*Loktanela* (Alphaproteobacteria, Rhodobacteraceae)	6.1	*Rivularia* (Cyanobacteria, sub IV, fam II)	9.8
*Phormidium* (Cyanobacteria, sub III, fam I)	3.1	*Arthrospira* (Cyanobacteria, sub III, fam I)	4.4
*Jannaschia* (Alphaproteobacteria, Rhodobacteraceae)	2.8	*Phormidium* (Cyanobacteria, sub III, fam I)	2.3
*Roseovarius* (Alphaproteobacteria, Rhodobacteraceae)	2.4	Unass. genus 1 (Cyanobacteria, sub I, fam I)	1.0
*Wenxinia* (Alphaproteobacteria, Rhodobacteraceae)	2.2	Unass. genus 2 (Cyanobacteria, sub I, fam I)	0.8
*Porphyrobacter* (Alphaproteobacteria, Erythrobacteraceae)	2.0	*Spirulina* (Cyanobacteria, sub III, fam I)	0.7
**V3V4–DNA**	**%**	**V3V4–RNA**	**%**
Unass. genus 1 (Cyanobacteria, sub III, fam I)	28.3	Unass. genus 1 (Cyanobacteria, sub III, fam I)	30.9
*Coleofasciculus* (Cyanobacteria, sub III, fam I)	9.1	*Coleofasciculus* (Cyanobacteria, sub III, fam I)	16.0
*Phormidium* (Cyanobacteria, sub III, fam I)	7.0	*Lyngbya* (Cyanobacteria, sub III, fam I)	14.3
Unass. genus 2 (Cyanobacteria, sub III, fam I)	6.6	Unass. genus 2 (Cyanobacteria, sub III, fam I)	12.9
Unass. genus (Chloroflexi, Anaerolineaceae)	3.9	*Rivularia* (Cyanobacteria, sub IV, fam II)	12.2
*Loktanela* (Alphaproteobacteria, Rhodobacteraceae)	2.5	*Phormidium* (Cyanobacteria, sub III, fam I)	4.4
*Maribacter* (Bacteroidetes, Flavobacteriaceae)	1.9	*Trichodesmium* (Cyanobacteria, sub III, fam I)	1.2
Unass. genus (Bacteroidetes, Flavobacteriaceae)	1.5	Unass. genus 1 (Cyanobacteria, sub I, fam I)	1.0
Unass. genus (Chloroflexi, Caldilineaceae)	1.3	*Nannocystis* (Deltaproteobacteria, Nannocystaceae)	0.5
Unass. genus (Bacteroidetes, Saprospiraceae)	1.2	Unass. genus (Cyanobacteria, sub IV, fam II)	0.4

## Discussion

In order to understand the ecology of the communities of microbial ecosystems more than just one snapshot in time and space is required. Previous molecular genetic investigations of these microbial mats^22^ were hampered by under-sampling, prohibiting statistical relevant sample numbers, and by short sequence reads.

The present study demonstrates that for the investigated coastal microbial mats, the previously determined heterogeneity in community composition^23^ might not give the full picture of what happens to the community as a whole, taking into account the resident as well as the active fraction of the community. Cluster analysis in combination with PERMANOVA testing of significance showed that temporal variations in the bacterial diversity were negligible within the 24-h sampling period. The other variables (nucleotide source or variable region sequenced) did not affect this conclusion. Not even the RNA fraction revealed any temporal variations in the active community although it may be expected that growth and activity of different functional groups of microorganisms in the community might have shown day-night variations, for instance, under control of a circadian clock. This was obviously not the case in the investigated mats and this justifies the conclusion that the active microorganisms required the synthesis of ribosomes throughout the day-night cycle. Distinct diel variations in the active bacterial fraction have been observed in open waters^24^. These variations were more likely associated with the continuous dynamics of water movement such as upwelling and tide related currents that may introduce other microorganisms and therefore alter the community composition. In microbial mats in- and export of microorganisms is low and this results in the formation of a stable community composition. The discovery that the active community did not change over a day-night cycle is new, but may be understood considering the different metabolic processes that are constitutive in active microorganisms and therefore continuously require ribosomes. It is now clear from our investigation that there is limited spatial heterogeneity, which is in agreement with DGGE results variation in the community composition of these coastal microbial mats^23^, at least not when the sampling is limited to the relative small area chosen in this study. The 24-h sampling strategy is obviously not required for the study of the microbial community composition in coastal microbial mats, since the active microbial community did not change during a day-night cycle. We considered these samples as replicates for statistical analyses.

### Comparison of the two variable regions of the 16S rRNA gene

The 16S ribosomal RNA gene contains several highly variable and more conserved regions. Sanger sequencing allowed for high quality sequencing of up to 1000 nucleotides albeit at low throughput and focused on near full length sequencing of the 16S rRNA gene. Current high throughput sequencing technologies however are optimized for short read lengths and focus on one or more of the nine hypervariable regions^25^ or tandem pairs thereof. An optimal sequencing strategy should be defined as using truly universal, unbiased primers, amplifying a region that provide 100% coverage of the extant diversity and can rely on a complete reference database for taxonomic identification. However, this utopia is still far from reality and researchers rely on comparative studies and empirical data to make their choices. We compared two primer sets that amplify two tandem pairs of hypervariable regions; the V1-V3 and the V3-V4 hypervariable regions. One notable observation was that these pairs gave different community compositions. Although the major players were similar and both primer sets predicted ~60% of Cyanobacteria in the DNA fraction, the relative abundance of Proteobacteria and Bacteroidetes differed between the DNA and RNA fractions (Fig. 2A). Also Chloroflexi, Planktomycetes and Actinobacteria were more represented in the V3-V4 dataset in agreement with a previous study in which similar mats were studied by 454 pyrosequencing of the hypervariable V6 region^22^. The V3-V4 data set also predicted a higher Shannon diversity (4.9 versus 4.6 in the V1-V3 set) and a slightly better coverage at the genus level. Cai, *et al*.^26^ conducted a broad comparative study to the effect of choice of primer sets on the derived community composition. These authors compared short amplicons (<500 nt: V1-V2, V3-V4, V5-V6, and V7-V9) with long amplicons (723–795 nt: V1–V4, V3–V6, and V5–V9) and concluded that the V3-V4 region performed best based on little bias, and high genus coverage. However, other studies reported that, for example, the family of the Enterobacteriaceae and the Clostridiales were poorly resolved when using the V3-V4 primers^27^. Thus, different primer sets are promoted by different ambassadors but none of these are yet free of bias. For future experimentations we recommend the V3-V4 primer set because in our hands it provided a higher resolution at the genus level.

### Probing the active population

Several studies compared DNA with RNA based sequencing strategies to infer about the active microbial fraction in various environments such as soil, sediment, plankton^28–32^. The main assumption behind these comparisons is that RNA is retrieved only from active organisms. Moreover, the DNA pool may also consist of stable extracellular DNA, i.e. DNA from lysed cells that may have been accumulated over days, weeks, months or longer^33, 34^ and from cells that are still intact but inactive (e.g. dormant cells and spores) which may lead to an over-representation of certain species in the derived community composition. For stable communities one could argue that the extracellular DNA pool represents the actual community and would not be different when rRNA is used. Only in a highly dynamic system, these stable pools of DNA may lead to a misrepresentation of the actual diversity. RNA molecules are less stable mainly due to two reactive hydroxyl groups in the ribose backbone and to the presence of ribonucleases in the cells and environment^35^. A linear relation between rRNA production and cell activity however is not straight forward. Blazewicz and coworkers provided various examples where ribosomal RNA pools did not necessary accurately reflect growth rate or growth state and high numbers of ribosomes and thus ribosomal RNA may even occur in dormant cells^36^. Currently, it is unknown how much the presence of extracellular DNA affects the estimation of the resident community but we also do not exactly know whether, in the RNA fraction, the ribosomal synthesis translates in 1:1 into an organism’s activity. It is possible that in the rRNA pool the activity of some organisms deviates the measured outcome from the actual activity and diversity. Removal of extracellular DNA is difficult and not straight forward and it may affect living cells. The relationship of rRNA and activity is also not straight forward and there may be considerable differences between different community members. Therefore, the most pragmatic approach is to maintain the extant (DNA) versus active (RNA) division but interpret the data cautiously.

In this study, all classified phyla were recovered from both the DNA and RNA fractions with the exception of some rare candidate phyla that were also not equally distributed over the replicates. At the genus level, 8% (V1-V3) to 8.5% (V3-V4) fewer genera were found in the RNA fraction relative to the DNA fraction although in general these only consisted of the low abundant genera. The largest difference between the RNA and DNA fractions could be explained by cyanobacterial reads that made up 94% of the total number of reads in the RNA and 60% in the DNA fraction. Also at the genus level the Cyanobacteria were more represented in the RNA fraction. However, Proteobacteria contributed less than 0.01% of the total active community and were not found in all replicates

Given the macro and microscopic visual dominance of Cyanobacteria and their important role as primary producers in microbial mats, it is not surprising to see that they comprise the majority of the RNA derived gene sequences. Nevertheless, the actual numerical dominance of Cyanobacteria in ‘Cyanobacterial’ mats has been questioned by several authors^13, 37, 38^. In high Arctic hypersaline springs microbial mats dominated by Cyanobacteria (~20% of total diversity) in the DNA fraction appeared to be totally absent in the RNA fraction^38^. This was attributed to the dormancy of the Cyanobacteria at the time of sampling. Although sampling of the mats in our study was rather late in the season (early November) when development of the mats had slowed down, Cyanobacteria still outnumbered almost all other functional groups of microorganisms in this microbial mat.

The active Cyanobacteria consisted mainly of non-heterocystous filamentous species of Subsection III, Family I, which comprises the abundantly present genera such as *Coleofasciculus* (previously assigned as *Microcoleus chthonoplastes*), *Lyngbya (L. aestuarii*), and *Phormidium*, although the majority of sequences belonged to currently unassigned genera within this family. Subsection IV Cyanobacteria (heterocystous N_2_-fixing Cyanobacteria) are 7-fold more abundant in the RNA fraction (11.5%) compared to the DNA fraction (1.7%), whereas Subsection III cyanobacteria are slightly less represented in the RNA fraction than in the DNA fraction (94% RNA - 85% DNA). Unicellular Cyanobacteria of Subsection I remain at a similar relative abundance (~3%) in the DNA and RNA fractions.

Cyanobacteria are, together with diatoms, the most important and dominant primary producers of the coastal microbial mat ecosystem^39^. Previous studies in similar coastal mats using microscopy, revealed that unicellular Cyanobacteria, *Synechococcus* sp., and filamentous *Spirulina* sp. are often observed in the early stages of the mat formation. The majority of cyanobacterial genera found in the RNA fraction is member of the subsection III, family I group, in accordance with previous studies^22, 40^. Unicellular Cyanobacteria are frequently observed using microscopy, but in our dataset the majority of the retrieved Cyanobacteria is filamentous. It is possible that the chosen primers are not appropriate for unicellular Cyanobacteria, but microscopic observations confirmed the vast dominance of filamentous Cyanobacteria. The data show predominance of *Coleofasciculus*, *Rivularia*, *Lyngbya* and, to some extent, *Spirulina*. *Coleofasciculus* was previously described as one of the most abundant organisms in the intermediate station by other studies^22, 41^. In the present study, *Coleofasciculus* is amongst the most abundant Cyanobacterial group in the active fraction, which probably results in the fact that this cyanobacterium is amongst the first colonizers of this system presumably having an important role on the survival of the mat during the winter time.

A curiosity in our dataset is *Trichodesmium*, a planktonic, gas-vacuolated, N_2_-fixing cyanobacterium found in tropical and subtropical areas of the ocean^42^. This cyanobacterium was among the top 10 of the active population of the microbial mats when considering the V3-V4 region (Table 2). The presence of the previous described planktonic *Trichodesmium* species itself in microbial mats is not plausible, but instead may point towards a close relative of *Trichodesmium* that has adapted to benthic life in a microbial mat.

Using pyrosequencing technology to sequence the V6 16S rRNA region, Cyanobacteria were found to be an important component of the microbial community but Proteobacteria clearly dominated the resident fraction of the microbial mat^22^. The dominance of Proteobacteria can be understood considering their versatile metabolism as aerobes or facultative aerobes. Some representatives are even chemolithotrophic or phototrophic^43^. This class is important for several metabolic processes in microbial mats such as ammonium oxidation but also contributes to biogeochemical cycles of carbon, sulfur and nitrogen. In Bolhuis and Stal’s study^22^, the most abundant organisms retrieved belonged to proteobacterial phyla. In the samples analyzed in this study, we see that Cyanobacteria dominate both the DNA and RNA fractions of the microbial mat, followed by Proteobacteria and Bacteroidetes. When looking at the class level, Alphaproteobacteria were the most abundant Proteobacteria in the RNA dataset (0.0127%), followed by mainly sulfate-reducing Deltaproteobacteria (0.0123%). In the DNA fraction, however, Gammaproteobacteria were the most abundant at 0.075%, followed by Alphaproteobacteria at 0.066% while the contribution of the Gammaproteobacteria to the RNA fraction was only 0.009%. The dominant gammaproteobacterial orders found in this study, Alteromonadales, Oceanospirillales and Vibrionales are almost exclusively of marine origin^44^. This may explain their lower relative activity and under brackish conditions they may have entered a state of dormancy lasting until the mats are again exposed to full marine salinity. Several organisms belonging to the Alpha and Gammaproteobacteria are known to be involved in anaerobic sulfate and sulfur reduction^44^. Throughout the year, the sulfur cycle is important in coastal microbial mats and it is therefore expected that Alpha-, Gamma- and Deltaproteobacteria are active at any time of the year.

Betaproteobacteria are present at very low levels in the RNA (0.0004%) and DNA (0.003%) fraction (Fig. 2C). Some of the organisms belonging to this group perform functions related to the sulfur and nitrogen cycles. These cycles are among the most important cycles in marine microbial mats and therefore we expect organisms that carry out these biogeochemical processes to be active throughout the year. Actually, Betaproteobacteria are wide spread in freshwater- and terrestrial environments and are found more often in the fresh water dominated mats of Schiermonnikoog that are found close to the dunes^22^. An exception to the freshwater life style of Betaproteobacteria is a rare species that had a short winter bloom in the Arctic Ocean^45^. The microbial mat investigated in this study is mainly influenced by brackish water because it receives occasional seawater at spring tides and fresh water from rain or upwelling ground water. This difference in salt concentration may be too hostile for Betaproteobacteria and may explain their low abundance in the RNA and DNA fraction.

Actinobacteria were not among the key players in the investigated microbial mats, which seems in contradiction with the observation made in 2009 by Bolhuis and Stal^22^. Nevertheless, the present study agrees with the previous one on the other groups of microorganisms that were identified as major players in the Schiermonnikoog microbial mats. Despite the differences in relative community composition derived from the DNA fraction between these two studies it can be concluded that both identified the same major players in the mat, regardless the different techniques, the different times of sampling, and the different 16S rRNA regions that were sequenced. The differences can be attributed to the natural evolution of the microbial mat and to the applied sampling strategy.

For the Proteobacteria, the largest differences between the two selected 16S rRNA regions may be attributed to the choice of the primer. Alphaproteobacteria dominated the V1-V3 dataset while the V3-V4 dataset was dominated by Gammaproteobacteria at the DNA level. Nevertheless, the V3-V4 region revealed a more diverse representation of the proteobacterial class, which is in accordance with previous results in various microbial mats and in accordance with the expected diversity of sulfide oxidizing Gamma- and Alphaproteobacteria and sulfate-reducing Deltaproteobacteria^1, 46^. The class Cytophaga was more abundant in the RNA fraction, while Flavobacteria dominated the DNA fraction. Both Cytophaga and Flavobacteria are known to degrade organic matter suggesting that at time of sampling, Cytophaga are the most likely candidates performing this important recycling function.

We conclude that: 1) using 16S rRNA sequence analysis there is no change in the activity of the different groups of microorganisms in the microbial mat during a 24-h day; 2) Cyanobacteria are the main players in this microbial mat at the time of sampling. They dominate both the active (RNA) and resident (DNA) fractions; 3) the nucleotide source (RNA or DNA) affects the outcome of the microbial diversity analysis. Hence, there is a discrepancy between the organisms present in the microbial mat and those that are actually active. The resident and active microbial communities are significantly different. This different community composition translates in the success of the whole ecosystem which confirms the cooperation between all organisms, active and non-active; 4) The application of different approaches, such as the analysis of both the DNA and RNA fractions, the sequencing of more than one region of the 16S rRNA, and statistical relevant sampling strategy (sufficient replicates, sampling of larger area, 24-h sampling), is currently the best way of avoiding biases inherent of these molecular diversity studies. The region V3-V4 of the 16S rRNA gene proved to be the most suitable to uncover the diversity of this microbial mat ecosystem.

## Materials and Methods

### Sampling site and sampling procedure

Samples were taken from a coastal microbial mat on the North Sea beach of the Dutch barrier island Schiermonnikoog (53°29.410′ N, 6°,08.356′ E) on 6 and 7 November 2013 at 4-h intervals during a 24-h period (at 18:00, 22:00, 06:00, 10:00, 14:00, and again at 18:00). Sampling at 02:00 was skipped because of the inaccessibility of the site. The targeted microbial mat was of the previously described as “intermediate type”, located between the tidal zone and the vegetated freshwater zone^23^. At each time point 10 subsamples were taken randomly within a 15 × 15 m area with a sterile 10-ml syringe, of which the top was removed in order to extract a core of ~10 mm in diameter. The top 5 mm of each core of each the sample was split in two halves along the vertical axis, each covering the full 5 mm of mat layers, for respectively DNA and RNA extraction. For DNA extraction, the samples were put in in sterile 5-ml cryotubes (VWR International, Pennsylvania), snap frozen in liquid nitrogen and put on dry ice for transportation to the lab where they were stored at −80 °C until processing. Samples for RNA extraction were first mixed with 2.5 ml of LifeGuard™ Soil Preservation Solution (MOBIO, Carlsbad, CA, USA) to prevent RNA degradation.

### Nucleic acid extraction and copy DNA synthesis

DNA was independently extracted from each of the 60 samples using the PowerSoil® DNA isolation kit (MOBIO, Carlsbad, CA, USA) according to the manufacturer’s instructions. DNA yield and quality was checked by NanoDrop 1000 (NanoDrop, Wilmington, DE, USA) and by agarose gel electrophoresis. For further processing and standardization, the DNA concentration was adjusted to 20 ng µl^−1^. After DNA quantification, the 10 samples taken at one time point were pooled resulting in 6 samples each representing a different point an time and different part of the microbial mat. RNA was extracted from ~2 g wet weight of microbial mat using the PowerSoil® RNA extraction kit (MOBIO, Carlsbad, CA, USA) according to the manufacturer’s instructions. Total RNA yield and quality was measured using the Agilent 2100 Bioanalyzer (Agilent Technologies, Santa Clara, CA, USA) and samples with a RNA Integrity Number (RIN) lower than 6 were excluded from further analysis. RNA concentrations were adjusted to 50 ng µl^−1^, whereafter the 10 subsamples were pooled. Residual DNA in the RNA fraction was removed using the TURBO DNA-free™ DNAse kit (Ambion/Life Technologies - Thermo Fisher Scientific, USA) and removal was confirmed by 16S rRNA gene PCR amplification using a DNA dependent polymerase (ThermoFisher Scientific, Pittsburgh, PA, USA) with universal bacterial primers B8F^47^ and U1492R^48^ and subsequent agarose gel electrophoresis (1.5% agarose gel). From the DNA free RNA fractions, copyDNA (cDNA) was synthesized using SuperScript® III Reverse Transcriptase (ThermoFisher Scientific) according to the manufacturer’s instructions. Successful reverse transcription and synthesis of cDNA was validated by PCR analysis as described above.

The 6 DNA and 6 cDNA samples were submitted to BGI (Hong Kong, China) (http://www.genomics.cn/en/index) for bacterial 16S rRNA amplicon sequencing using the Illumina MiSeq platform. Two different regions of the bacterial 16S rRNA gene were targeted with the V1-V3 primer set (V1 primer – B8F, 5′-AGAGTTTGATCCTGGCTCAG-3′ and V3 primer – 534 R, 5′-ATTACCGCGGCTGCTGG-3′)^49^ and the V3-V4 primer set (V3 primer – 338 F, 5′-ACTCCTACGGGAGGCAGCAG-3′^50^ and V4 primer – 806 R, 5′-GGACTACHVGGGTWTCTAAT-3′^51^). Sequencing insert library construction, barcoding and amplicon sequencing were done by BGI. Raw sequences in fastq format were submitted to the Sequence Read Archive, https://www.ncbi.nlm.nih.gov/sra, under the BioProject accession number SUB2291354.

### Bioinformatics and statistical analysis

Microbial community analysis was largely done using algorithms and programs run from the QIIME pipeline^52^. Raw Illumina PE300 reads were paired and pre-processed using PEAR^53^. A quality filter step was used to filter out reads with ambiguous bases (Ns), *i.e*. reads composed by less than 250 nucleotides, or reads of which the average quality scores, using a sliding window of 40 bp, dropped below a satisfactory threshold of 25 Phred. Reads were further processed with Vsearch 1.1.3 (https://github.com/torognes/vsearch)^54^, in order to remove chimeras prior to performing de novo clustering into OTUs at 95% sequence identity. Rare OTUs with less than 75 reads in the combined amplicon data were discarded. The cluster centroid for each OTU was chosen as the OTU representative sequence. The taxonomic assignment of the representative sequences was carried out using the RDP Bayesian Classifier^55^ against the SILVA SSU non-redundant database (version 111 release) that was pre-aligned and clustered at 97% identity, and using a consensus confidence threshold of 0.8. The RDP classifier was then used to assign OTUs, using the fifth and sixth taxonomic level wherever possible, which in most cases corresponded to family and genus ranks. To determine the suitable sequencing depth that covers the extant microbial diversity for each time point, a rarefaction table was calculated for each sample. Microbial diversity between samples (beta-diversity) and statistical significance was determined by Non-metric multidimensional scaling using a Bray-Curtis dissimilarity matrix and PERMANOVA using the ADONIS functions of the vegan R Package^56^. Venn diagram was created by calculating the number of shared and unique genera in the different datasets in Excel.

## References

[1] van Gemerden H (1993). Microbial mats: A joint venture. Mar Geol.

[2] McDougald D, Rice SA, Barraud N, Steinberg PD, Kjelleberg S (2011). Should we stay or should we go: mechanisms and ecological consequences for biofilm dispersal. Nat Rev Microbiol.

[3] Cantrell SA, Duval-Perez L (2012). Microbial mats: an ecological niche for fungi. Front. Microbiol..

[4] Cuadrado DG, Carmona NB, Bournod C (2011). Biostabilization of sediments by microbial mats in a temperate siliciclastic tidal flat, Bahia Blanca estuary (Argentina). Sediment Geol.

[5] Ward DM, Ferris MJ, Nold SC, Bateson MM (1998). A natural view of microbial biodiversity within hot spring cyanobacterial mat communities. Microbiol Mol Biol Rev.

[6] Allen MA, Neilan BA, Burns BP, Jahnke LL, Summons RE (2010). Lipid biomarkers in Hamelin Pool microbial mats and stromatolites. Org. Geochem..

[7] Dijkman NA, Boschker HTS, Stal LJ, Kromkamp JC (2010). Composition and heterogeneity of the microbial community in a coastal microbial mat as revealed by the analysis of pigments and phospholipid-derived fatty acids. J Sea Res.

[8] Pages A (2014). Diel fluctuations in solute distributions and biogeochemical cycling in a hypersaline microbial mat from Shark Bay, WA. Mar Chem.

[9] Ferris MJ, Ruff-Roberts AL, Kopczynski ED, Bateson MM, Ward DM (1996). Enrichment culture and microscopy conceal diverse thermophilic *Synechococcus* populations in a single hot spring microbial mat habitat. Appl Environ Microbiol.

[10] Wade W (2002). Unculturable bacteria–the uncharacterized organisms that cause oral infections. J R Soc Med.

[11] Stewart EJ (2012). Growing unculturable bacteria. J Bacteriol.

[12] Pedrós-Alió C (2006). Marine microbial diversity: can it be determined?. Trends Microbiol.

[13] Sogin ML (2006). Microbial diversity in the deep sea and the underexplored “rare biosphere”. Proc Natl Acad Sci USA.

[14] Qin J (2010). A human gut microbial gene catalogue established by metagenomic sequencing. Nature.

[15] Chakravorty S, Helb D, Burday M, Connell N, Alland D (2007). A detailed analysis of 16S ribosomal RNA gene segments for the diagnosis of pathogenic bacteria. J Microbiol Methods.

[16] Dell’Anno A, Fabiano M, Duineveld GCA, Kok A, Danovaro R (1998). Nucleic acid (DNA, RNA) quantification and RNA/DNA ratio determination in marine sediments: comparison of spectrophotometric, fluorometric, and HighPerformance liquid chromatography methods and estimation of detrital DNA. Appl Environ Microbiol.

[17] Josephson KL, Gerba CP, Pepper IL (1993). Polymerase chain reaction detection of nonviable bacterial pathogens. Appl Environ Microbiol.

[18] Masters CI, Shallcross JA, Mackey BM (1994). Effect of stress treatments on the detection of Listeria monocytogenes and enterotoxigenic Escherichia coli by the polymerase chain reaction. J Appl Bacteriol.

[19] Klappenbach JA, Dunbar JM, Schmidt TM (2000). rRNA operon copy number reflects ecological strategies of bacteria. Appl Environ Microbiol.

[20] Birch L, Dawson CE, Cornett JH, Keer JT (2001). A comparison of nucleic acid amplification techniques for the assessment of bacterial viability. Lett Appl Microbiol.

[21] Keer JT, Birch L (2003). Molecular methods for the assessment of bacterial viability. J Microbiol Methods.

[22] Bolhuis H, Stal LJ (2011). Analysis of bacterial and archaeal diversity in coastal microbial mats using massive parallel 16S rRNA gene tag sequencing. ISME J.

[23] Bolhuis H, Fillinger L, Stal LJ (2013). Coastal microbial mat diversity along a natural salinity gradient. PloS one.

[24] Gilbert JA (2010). The taxonomic and functional diversity of microbes at a temperate coastal site: a ‘multi-omic’ study of seasonal and diel temporal variation. PloS one.

[25] Yarza P (2014). Uniting the classification of cultured and uncultured bacteria and archaea using 16S rRNA gene sequences. Nat Rev Microbiol.

[26] Cai L, Ye L, Tong AH, Lok S, Zhang T (2013). Biased diversity metrics revealed by bacterial 16S pyrotags derived from different primer sets. PloS one.

[27] Jovel J (2016). Characterization of the gut microbiome Using 16S or shotgun metagenomics. Front. Microbiol.

[28] Moeseneder MM, Winter C, Herndl GJ (2001). Horizontal and vertical complexity of attached and free-living bacteria of the eastern Mediterranean Sea, determined by 16S rDNA and 16S rRNA fingerprints. Limnol Oceanogr.

[29] Moeseneder MM, Arrieta JM, Herndl GJ (2005). A comparison of DNA- and RNA-based clone libraries from the same marine bacterioplankton community. FEMS Microbiol Ecol.

[30] Mills HJ, Martinez RJ, Story S, Sobecky PA (2005). Characterization of microbial community structure in Gulf of Mexico gas hydrates: comparative analysis of DNA- and RNA-derived clone libraries. Appl Environ Microbiol.

[31] Gentile G (2006). Study of bacterial communities in Antarctic coastal waters by a combination of 16S rRNA and 16S rDNA sequencing. Environ Microbiol.

[32] Rodriguez-Blanco A, Ghiglione JF, Catala P, Casamayor EO, Lebaron P (2009). Spatial comparison of total vs. active bacterial populations by coupling genetic fingerprinting and clone library analyses in the NW Mediterranean Sea. FEMS Microbiol Ecol.

[33] Dell’Anno A, Danovaro R (2005). Extracellular DNA plays a key role in deep-sea ecosystem functioning. Science.

[34] Nielsen KM, Johnsen PJ, Bensasson D, Daffonchio D (2007). Release and persistence of extracellular DNA in the environment. Environ Biosafety Res.

[35] Li Y, Breaker RR (1999). Kinetics of RNA degradation by specific base catalysis of transesterification involving the 2‘-hydroxyl group. J Am Chem Soc.

[36] Blazewicz SJ, Barnard RL, Daly RA, Firestone MK (2013). Evaluating rRNA as an indicator of microbial activity in environmental communities: limitations and uses. ISME J.

[37] Ley RE (2006). Unexpected diversity and complexity of the Guerrero Negro hypersaline microbial mat. Appl Environ Microbiol.

[38] Lay CY (2013). Defining the functional potential and active community members of a sediment microbial community in a high-arctic hypersaline subzero spring. Appl Environ Microbiol.

[39] Stal LJ (1995). Physiological ecology of cyanobacteria in microbial mats and other communities. New Phytol.

[40] Severin I, Stal LJ (2008). Light dependency of nitrogen fixation in a coastal cyanobacterial mat. ISME J.

[41] Severin I, Acinas SG, Stal LJ (2010). Diversity of nitrogen-fixing bacteria in cyanobacterial mats. FEMS Microbiol Ecol.

[42] Capone DG, Zehr J, Paerl H, Bergman B, Carpenter EJ (1997). Trichodesmium, a globally significant marine cyanobacterium. Science.

[43] Kersters, K. *et al*. In The Prokaryotes. An electronic handbook on the biology of bacteria: ecophysiology, isolation, identification, and applications (eds A. Balows *et al*.) 3–37 (Springer-Verlag KG, Berlin, Germany, 2003).

[44] Yilmaz P, Yarza P, Rapp JZ, Glöckner FO (2015). Expanding the world of marine bacterial and archaeal clades. Frontiers in Microbiology.

[45] Alonso-Saez L (2014). Winter bloom of a rare betaproteobacterium in the Arctic Ocean. Front Microbiol.

[46] Bolhuis H, Cretoiu MS, Stal LJ (2014). Molecular ecology of microbial mats. FEMS Microbiol Ecol.

[47] Edwards U, Rogall T, Blocker H, Emde M, Bottger EC (1989). Isolation and direct complete nucleotide determination of entire genes. Characterization of a gene coding for 16S ribosomal RNA. Nucleic Acids Res.

[48] Turner S, Pryer KM, Miao VP, Palmer JD (1999). Investigating deep phylogenetic relationships among cyanobacteria and plastids by small subunit rRNA sequence analysis. J Eukaryot Microbiol.

[49] Muyzer G, de Waal EC, Uitterlinden AG (1993). Profiling of complex microbial populations by denaturing gradient gel electrophoresis analysis of polymerase chain reaction-amplified genes coding for 16S rRNA. Appl Environ Microbiol.

[50] Lane, D. J. In *Nucleic acid techniques in bacterial systematics* (eds E. Stackebrandt & M. Goodfellow) 115–175 (John Wiley and Sons, 1991).

[51] Caporaso JG (2011). Global patterns of 16S rRNA diversity at a depth of millions of sequences per sample. Proc Natl Acad Sci USA.

[52] Caporaso JG (2010). QIIME allows analysis of high-throughput community sequencing data. Nat Methods.

[53] Zhang J, Kobert K, Flouri T, Stamatakis A (2014). PEAR: a fast and accurate Illumina Paired-End reAd mergeR. Bioinformatics.

[54] Rognes, T., Flouri, T., Nichols, B., Quince, C., & Mahé, F. VSEARCH: a versatile open source tool for metagenomics. *Peer. J*. **4**, e2584, ISO 690 (2016). 10.7717/peerj.2584 PMC5075697 27781170

[55] Wang Q, Garrity GM, Tiedje JM, Cole JR (2007). Naive Bayesian classifier for rapid assignment of rRNA sequences into the new bacterial taxonomy. Appl Environ Microbiol.

[56] Dixon P (2003). VEGAN, a package of R functions for community ecology. J Veg Sci.

